# Anatomical Atlas; Illustrative of the Structure of the Human Body

**Published:** 1844-10

**Authors:** 


Art. XII.—*Anatomical Atlas; illustrative of the Structure of the Human Body.* By Henry H. Smith, M.D., &c.; under the supervision of William E. Horner, M. D., &c. Philadelphia: Lea & Blanchard. 1844. Part III.

This number of the Anatomical Atlas is occupied with a description of the *organs of Digestion and Generation*, which are exhibited in 191 figures, of the same beautiful execution which characterized the first parts of the work. We are glad to find that the Atlas is to be continued. Every effort to extend a knowledge of anatomy deserves the encouragement of the profession. A more elegant work than the one before us could not easily be placed by a physician upon the table of his students.

